# Effectiveness of a cognitive behavioural therapy-based rehabilitation programme (Progressive Goal Attainment Program) for patients who are work-disabled due to back pain: study protocol for a multicentre randomised controlled trial

**DOI:** 10.1186/1745-6215-14-290

**Published:** 2013-09-11

**Authors:** Miriam N Raftery, Andrew W Murphy, Eamon O’Shea, John Newell, Brian E McGuire

**Affiliations:** 1 School of Psychology, National University of Ireland, Galway, Ireland; 2 Centre for Pain Research, National University of Ireland, Galway, Ireland; 3 Discipline of General Practice, National University of Ireland, Galway, Ireland; 4 Department of Economics, National University of Ireland, Galway, Ireland; 5 Irish Centre for Social Gerontology, National University of Ireland, Galway, Ireland; 6 HRB Clinical Research Facility and School of Mathematics, Statistics and Applied, Mathematics, National University of Ireland, Galway, Ireland

**Keywords:** Active rehabilitation, Back pain, Cognitive-behavioural therapy, Return to work

## Abstract

**Background:**

Psychologically informed rehabilitation programmes such as the Progressive Goal Attainment Program (PGAP) have the potential to address pain-related disability by targeting known psychological factors that inhibit rehabilitation progress. However, no randomised controlled trials of this intervention exist and it has not been evaluated in the Irish health service context. Our objective was to evaluate the clinical efficacy and cost-effectiveness of the PGAP in a multicentre randomised controlled trial with patients who are work-disabled due to back pain.

**Methods and design:**

Adult patients (ages 18 years and older) with nonmalignant back pain who are work-disabled because of chronic pain and not involved in litigation in relation to their pain were invited to take part. Patients were those who show at least one elevated psychosocial risk factor (above the 50^th^ percentile) on pain disability, fear-based activity avoidance, fatigue, depression or pain catastrophizing. Following screening, patients are randomised equally to the intervention or control condition within each of the seven trial locations. Patients allocated to the control condition receive usual medical care only. Patients allocated to the PGAP intervention condition attend a maximum of 10 weekly individual sessions of structured active rehabilitation in addition to usual care. Sessions are delivered by a clinical psychologist and focus on graded activity, goal-setting, pacing activity and cognitive-behavioural therapy techniques to address possible barriers to rehabilitation.

The primary analysis will be based on the amount of change on the Roland Morris Disability Questionnaire posttreatment. We will also measure changes in work status, pain intensity, catastrophizing, depression, fear avoidance and fatigue. Outcome measures are collected at baseline, posttreatment and 12-month follow-up. Health-related resource use is also collected pre- and posttreatment and at 12-month follow-up to evaluate cost-effectiveness.

**Discussion:**

This study will be the first randomized controlled trial of the PGAP in chronic pain patients and will provide important information about the clinical and cost effectiveness of the programme as well as its feasibility in the context of the Irish health service.

**Trial registration:**

Current Controlled Trials: ISRCTN61650533

## Background

Back pain constitutes a major health problem. In Ireland, between 13% and 36% of the population have chronic pain, with back pain being the most frequently reported site of pain [1,2]. Importantly, of those who consult their general practitioner (GP) for chronic pain, 60% to 80% still have pain and disability 1 year later [3,4]. The economic burden associated with chronic pain is also considerable. Recent research has estimated the cost of chronic pain at €5.34 billion, or 2.86% of gross domestic product per year in Ireland [5], with a large proportion of these costs being attributable to wage replacement for those unable to work because of back pain. Over the past decade, research has consistently highlighted the importance of psychological factors in the development and maintenance of back pain disability [6]. Specifically, fear avoidance beliefs [7], low mood [8] and a cognitive process known as catastrophizing [9] are consistently related to poor outcome [10,11]. There is evidence that these variables may play an important role in maintaining disability beyond the expected recovery time for soft-tissue injury and may be significant barriers to activity involvement and successful rehabilitation [12].

Although some reviews have shown only modest results for the success of interventions that target psychosocial risk factors [10,13], improvements in patient outcomes have been demonstrated when subgrouping has been used to guide treatment [14,15]. This subgrouping, or ‘matching’, of patients to different treatment options on the basis of psychological risk factors [14] has demonstrated that psychologically informed management can result in both health and economic benefits.

One such psychologically informed pain rehabilitation programme is the Progressive Goal Attainment Program (PGAP) [12,16]. PGAP and its predecessor the Pain Disability Prevention (PDP) program (under which name this trial was originally registered) were designed in Canada to reduce long-term disability arising from pain and other health conditions. PGAP is a manual-driven, 10-week rehabilitation programme designed to target evidence-based psychosocial risk factors for pain-related disability. These risk factors include fear-avoidance beliefs, pain catastrophizing, perceived injustice and depression. One of the most important components of this programme is activity mobilization. It has been acknowledged [12], however, that the predominately physical conceptualization of activity mobilisation may account for the relative lack of rehabilitation success for some patients [17]. Therefore, the role of psychological factors in facilitating and impeding activity mobilisation may have been underestimated. It has been argued [12] that activity mobilization is a form of behaviour change, one that involves a complex interplay among several psychological variables. The incorporation of psychological techniques to promote activity mobilisation is the basis for the PGAP.

The PGAP has proven efficacy in reducing disability and promoting return to work among people with work-related musculoskeletal pain [16] and whiplash injury [18]. However, there has not yet been a randomized controlled trial of PGAP in a chronic pain population. Thus, there is a need for an RCT study in which this rehabilitation programme (plus usual medical treatment) is compared to usual medical treatment alone. In addition, the PGAP has been developed in the context of a highly systematic and structured worker’s compensation system in Canada, but Ireland does not have a formal worker’s compensation system or rehabilitation case management. There is also very little access to psychologically informed back pain interventions at a community level in Ireland. Most routine pain management is provided by primary care GPs and physiotherapists who work in the public and private sectors and generally offer generic treatment services. However, there are virtually no other community-based pain management services in Ireland. Ireland does not have a national policy on the management of pain, nor does it have any general practice guidelines for the management of pain [19]. In the absence of a systematic pain management infrastructure in the community, the evaluation of a service that could be provided over and above normal primary care provision would seem highly valuable.

The present study was designed to examine the effectiveness of this psychologically based rehabilitation programme with a view to bridging this gap in service provision. We also propose to conduct a qualitative study with those receiving PGAP and the primary referral sources to get feedback on the user-friendliness of the programme, since establishing the feasibility of the programme is a specific objective.

The rationale for an economic evaluation of the PGAP is evident when one looks at the costs of long-term pain and associated disability [2,20]. Even a small reduction in the number of people with chronic disability is expected to be highly cost-effective and to greatly reduce the personal burden on those affected by chronic pain [20,21]. The study proposed herein will include a detailed cost–benefit analysis of the intervention as well as an analysis of its cost-effectiveness in terms of any cost savings associated with reduced disability. This will include an evaluation of both direct and indirect costs.

In summary, the study described herein has the following aims: (1) to examine the feasibility of implementing a community-based psychological pain rehabilitation programme (PGAP) aimed at treating patients who show elevated psychosocial risk factors for long-term pain-related disability, (2) to assess the effectiveness of the programme in a randomised controlled trial comprising PGAP plus medical treatment as usual (MTAU) versus MTAU only, (3) to determine the costs and cost savings associated with the programme by utilising appropriate indicators of health-related costs, (4) to gather feedback about the programme from key stakeholders and (5) to determine whether any changes achieved as a result of the programme are maintained at 12-month follow-up.

## Methods and design

This PGAP study is a pragmatic, multicentre randomised controlled trial of a cognitive behavioural therapy–based active rehabilitation programme for patients who are work-disabled due to back pain.

### Setting

The trial intervention is delivered by clinical psychologists based in eight regions of the Republic of Ireland: Galway, Mayo, Sligo, Donegal, Limerick, Cork and Dublin. The coordinating centre is the Centre for Pain Research at the National University of Ireland, Galway (NUI Galway), involving the academic departments of psychology, general practice, economics and the HRB Clinical Research Facility Biostatistics Unit. The intervention programme, PGAP, is delivered by public sector clinical psychologists based in each region. However, trial management and data collection take place at the Centre for Pain Research at NUI Galway.

### Hypothesis

The primary objective of the present study is to evaluate whether patients with back pain who take part in the PGAP (intervention group) show a greater mean reduction in reported disability compared to those who receive MTAU only (control group). Secondary objectives are (1) to examine changes in work status postintervention among those in the intervention group compared with the change in those in the control group (2) to examine changes in psychological variables among those in the intervention group compared to those in the control group and (3) to examine the cost-effectiveness of the programme to assess whether the costs of treatment are less than the savings associated with better outcomes.

### Recruitment and eligibility

#### Recruitment

**Figure 1:**
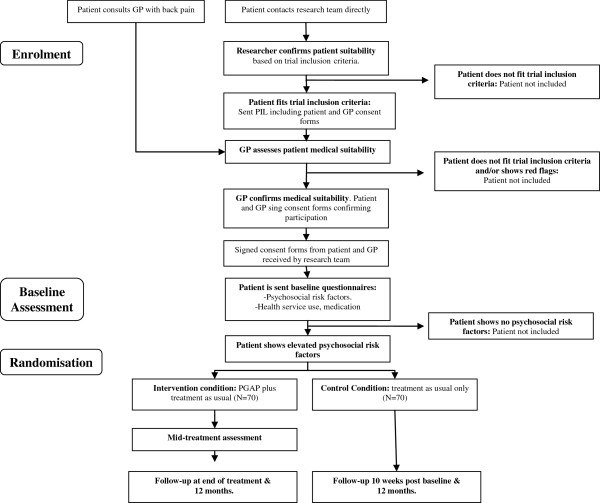
Flowchart of patient recruitment.

Potential participants are recruited directly from GPs and via self-referral (Figure 1). GP practices in Galway, Sligo, Limerick, Donegal, Cork, Mayo and Dublin were approached and informed about the study. The trial was also advertised in the local media at each trial location and within physiotherapy and primary care practices. These announcements offered patients the option to contact the research team directly if they were interested in taking part in the trial. Patients who contacted the research team directly were initially interviewed briefly over the phone by the researcher involved in the trial. Those who fit the initial inclusion criteria (see below) were given a brief explanation of what the trial involved and were then sent a detailed information leaflet explaining all aspects of the trial. The information leaflet also contained two consent forms, one to be signed by the patient and one to be signed by the patient’s GP. Patients were asked to bring this information leaflet and the consent form to their GPs. Each GP was asked to sign the consent form to confirm the patient’s medical suitability to take part in the trial.

Patients who are referred to the trial by their GP are given the information leaflet and consent form, as described above, by their GP at consultation. Both consent forms are then returned to the research team. When the research team receives the signed consent form from both patient and GP, formal assessment measures are then sent to the patient to screen for the presence of elevated psychosocial risk factors.

#### Inclusion criteria

Participants are patients with nonmalignant back pain 18 to 65 years of age who are not working or working no more than 50% of their usual work hours because of nonmalignant back pain. Back pain must be present for at least 6 weeks. GPs must confirm the presence of the painful condition and approve their patients’ participation in an activation programme.

#### Exclusion criteria

Patients are not eligible to participate in the trial if they meet any of the following exclusion criteria. (1) The GP confirms the presence of ‘red flags’ indicating serious pathology (for example, cancer, fracture, infection). (2) The patient has serious cognitive and psychiatric comorbidity. This criterion will include patients who have a diagnosis of a serious axis 1 or axis 2 mental health disorder as defined by the *Diagnostic and Statistical Manual of Mental Disorders, Fourth Edition* (DSM-IV). (3) The patient has a cognitive impairment that may affect the ability to engage in the programme (for example, Alzheimer disease, aphasia, stroke). The presence of these conditions will be confirmed by the patient’s GP. (4) The patient is currently receiving psychological management for pain. (5) The patient is involved in litigation in relation to the injury.

### Ethical approval

The final study protocol and the final version of the written informed consent form were approved by the NUI Galway Research Ethics Committee (Ref 09/NOV/10). Approval was also granted by individual hospital ethics committees at each trial site. The procedure set out in this protocol was designed to ensure that all persons involved in the trial abide by good clinical practice and by the ethical principles described in the current revision of the Declaration of Helsinki. Patients are required to sign a written consent form before being admitted to the clinical trial. The patient’s GP must also consent that the patient is medically suitable to take part in the research.

### Sample size

Following published guidelines [22], the sample size calculation was based on having 80% power at the 5% significance level to detect a difference in mean post-treatment improvement of 3 units or more on the Roland-Morris Back Pain Questionnaire between the control and intervention groups, assuming that the standard deviation of the improvement is 6.1 units in both groups [14]. In order to ensure sufficient power to detect such a difference, a sample size of 60 is needed for each arm. However, allowing for an attrition rate of 15% [16], and based on the experience of Sullivan *et al*. (personal communication MJ Sullivan, September 2007), we aim to include 140 participants (70 per group) in the study and expect to have complete data for about 120 participants following dropouts.

Screening of larger numbers of patients will be required to obtain a sample of 140. On the basis of recent research examining the prevalence of psychosocial risk factors among a general practice population, where at least one factor was found in 24% of respondents and up to 52% had a particular risk indicator, we expect that 20% to 50% of our sample frame will have one or more of the psychosocial risk factors [23].

### Screening measures

Patients who have returned written consent forms will be asked to complete a postal questionnaire pack containing measures of psychosocial risk factors and economic evaluation. Data will be collected from patients at baseline (prerandomisation), midtreatment (intervention group only, as this is part of the PGAP protocol), posttreatment (or at 10 weeks postbaseline for controls) and 12-month follow-up via postal questionnaire.

The primary outcome measure will be post-intervention changes in pain disability and work status post-treatment. Specific outcome measures were selected on the basis of the targeted psychosocial risk factors and on the Initiative on Methods, Measurement, and Pain Assessment in Clinical Trials (IMMPACT) recommendations for outcome measures for chronic pain clinical trials [24]. These measures include the following: (1) Roland-Morris Disability Questionnaire [25], (2) Chronic Pain Grade Questionnaire [26], (3) Short-Form McGill Pain Questionnaire [27], (4) Pain Catastrophizing Scale [28], (5) Fear-Avoidance Beliefs Questionnaire [29], (6) Hospital Anxiety and Depression Scale: Depression Subscale [30] and (7) Fear and Fatigue Questionnaire [12].

### Medication and health service use

Medication and health service use will be measured at baseline, posttreatment and follow-up for all patients. They will be measured using the pain version of the Client Service Receipt Inventory (CSRI) [31]. This will provide information on medication and health service resource use. The CSRI has been used widely in economic cost-of-illness studies, including studies of chronic pain [5,32,33], and has been validated as an accurate measure of frequency of health service use [34]. Medication use is likely to vary throughout the trial duration, and GPs will be allowed to vary medication as they see fit. However, change in medication use will be measured in post-treatment analysis. In cases of potential dual-use medication being taken by a patient, we will, with the patient’s consent, confirm with the prescribing GP if the medication was prescribed for pain or otherwise. Additional information will be gathered on (1) demographic variables (age, education, marital status); (2) pain-related information (duration of pain, number of pain sites, recurrence of pain, previous attendance at a pain management programme); and (3) details of any comorbid health conditions.

A patient is deemed suitable to go forward to the treatment allocation stage of the trial based on the presence of elevated psychosocial risk factors. This is defined as a score above the 50th percentile on any of the five risk factors targeted by the intervention: catastrophizing (Pain Catastrophizing Scale), fear of movement (Fear-Avoidance Beliefs Questionnaire), disability (Roland-Morris Disability Questionnaire), depression (Hospital Anxiety and Depression Scale) and fatigue (Fear and Fatigue Scale).

### Randomisation and treatment allocation

Eligible participants (that is, those who show elevated psychosocial risk factors) will be randomly assigned by computer-generated block randomisation to ‘intervention’ (PGAP sessions plus MTAU) or ‘control’ (MTAU only). We expect 20 participants to take part in the trial in each of the seven trial centres. Therefore, block randomisation with variable block sizes is used to minimize the occurrence of chance imbalance in allocation to treatment and control and to preserve unpredictability. The sequence of the different blocks is predetermined by an independent researcher not involved in the trial and concealed to all investigators. The blocks are generated separately for the different study sites so that stratification according to trial centre can be performed. It was decided to stratify on trial centre only for pragmatic reasons. Stratifying on additional variables, although useful, would have called for a larger sample. As each treating psychologist was seeing these trial patients in addition to their usual caseload, a limit was placed on the number of patients each clinician would be asked to see, thus limiting stratification.

### Progressive Goal Attainment Program

The PGAP is a 10-week one-to-one rehabilitation programme delivered in this trial by clinical psychologists. Each session is 1 hour long. The intervention was specifically designed to assist those individuals with thought patterns and behaviours which are known to increase risk for chronic disability. Patients are offered a maximum of 10 individual appointments with the PGAP psychologist. At their first appointment, patients receive an instructional DVD which orientates them to the PGAP and explains the importance of activity mobilization. Through the course of the treatment programme, the PGAP clinician assists the patient in identifying ways to increase activity involvement. Working with the PGAP clinician, the patient develops an activity schedule that is designed to keep him or her as active as possible. This may include household activities, running errands and social and recreational activities. A central element is a programme of regular walking. The patient’s spouse or partner is invited to participate in activity planning so that he or she is aware of the program that is being developed for the patient.

In the second phase of the program, the patient develops skills to overcome fears of reinjury and learns to monitor and modify self-defeating thinking that may accompany pain. The patient is taught methods of approaching physical activity in a way that minimizes worries and concerns about potential reinjury by using cognitive behavioural therapy strategies. Specifically, the programme addresses functional activity scheduling, activity pacing and psychological obstacles to progress such as fear of movement and reinjury, pain catastrophizing and the impact of negative or pessimistic thinking on emotional well-being and activity level. Finally, the patient learns communication skills and problem-solving strategies that will assist him or her in meeting the challenges brought on by the injury.

A total of 11 clinical psychologists employed in the public health service were trained to deliver the PGAP. This training involved attending a 2-day workshop delivered by the developer of the intervention (MJ Sullivan, McGill University, Montreal, QC, Canada). The psychologists involved in the trial are spread geographically throughout the Republic of Ireland and will see patients as part of this trial in their usual clinics.

Patients who have been randomly assigned to the intervention condition are contacted by the researcher involved in the trial to discuss their participation. An appointment is arranged with a PGAP psychologist who is geographically closest to the patient. Each patient is seen for a maximum of 10 hourlong one-to-one appointments with the PGAP psychologist. In parallel with these sessions, patients are free to access their GP and any other healthcare professional as usual. Patients are offered a maximum of 10 sessions with the clinical psychologist as part of the PGAP. Each patient is given a copy of the *Client Manual*, a workbook of exercises to be completed as part of the programme. The programme may be terminated earlier if the patient is able to return to work before attending all 10 sessions. Termination of treatment before the end of the 10 sessions will be at the discretion of each psychologist in consultation with the patient and patient’s GP.

### Control condition

Patients allocated to the control condition do not receive the PGAP. The patient is contacted by the researcher to explain that they have been allocated to the control condition and what this means. This gives the patient an opportunity to ask questions. This step is followed by written confirmation of the patient’s allocation to the control group. Patients in the control condition are free to access all healthcare services as usual. Patients who are randomised to the control condition will not be precluded from enrolling in a psychological pain management programme for the duration of this trial. If this happens, however, their data will be excluded from analysis. Participants in the control condition are sent assessment measures at baseline and at 10 weeks postbaseline. It is recognized that GP care will vary from individual to individual.

### Quantitative analysis

Patients participating in the PGAP trial are heterogeneous in terms of specific back pain problems, length of time in pain, history of attending pain management programmes and current medical management, including medication. Therefore, participating patients’ age, gender, marital status, length of time in pain, medical diagnosis (if any), number of pain sites, previous medical or therapeutic procedures and current medication is be gathered as potentially useful explanatory variables in all analyses.

The longitudinal change in the response variables of interest across treatment arms will be analysed using a linear mixed model to compare the change within individual participants (that is, before and after the intervention) and between the study arms (that is, comparing the cohort of patients who received the PGAP intervention to the control group participants), adjusting for baseline, treatment centre and patient characteristics as necessary. It will be assumed that missing data are missing at random, and the sensitivity to this assumption will be assessed using multiple imputations based on chained equations [35].

The selection of explanatory variables for inclusion in all final models will be based on a combination of clustering, tree-based methods and variable selection techniques applied to the complete cases and to the imputed data. All analyses will be carried out using R software [36] and SPSS software (SPSS Inc, Chicago, IL, USA) as necessary.

Model-checking will be performed using suitable model diagnostics and residual plots. Sensitivity analysis will be used to explore whether adherence to the intervention influences the effect of the intervention on the primary outcome.

### Qualitative analysis

Following the randomised controlled trial, qualitative research will be undertaken to explore the perceptions, views and experiences of patients and clinicians who participated and delivered the PGAP. Semistructured one-to-one interviews will be conducted with 15 participants and all clinicians within 3 months of completion of the PGAP. Participants will be purposively selected to ensure that a representative range of characteristics such as age and length of time in pain are taken into account. All interviews will be recorded and transcribed, and data analysis will be based on themes derived from the transcripts. NVivo, a qualitative software package (QSR International, Doncaster, VIC, Australia), will be used to facilitate data management and analysis.

### Economic analysis

Economic evaluation will consist of a standard cost–benefit analysis. An incremental analysis of cost-effectiveness will also be conducted to compare the costs and outcomes of the intervention to those in the control group. By establishing the costs of the programme and the associated health benefits, we can infer the cost-effectiveness of the intervention. Use of healthcare and social care services will be documented on the basis of questionnaire data obtained from the patients. We will collect two primary types of data on the economic impact of chronic pain: (1) the costs falling on healthcare and social care systems and (2) the costs falling on individuals and employers. Factors that contribute to costs include hospital visits, GP visits, medicines and other treatments. Intangible costs such as reduced quality of life will be extrapolated from international studies. The assessment of the opportunity cost of work will be based on days lost due to pain using information from the Central Statistics Office in Ireland. Family costs will also be documented and valuated in monetary terms. Costs to families will include time off from work to care for relatives with pain difficulties and direct costs such as fuel, use of cars and any treatments not covered by the state or insurance bodies. Unit costs will be calculated linking resource use to the best available cost data. In the case of hospital services, resource use will be assessed using diagnosis-related group unit costs. The cost of GP care will be calculated using the medical card capitation rates and the average charges for non–medical card patients. Costs to individuals and families will be calculated using average wage and other direct costs, such as travel costs. Uncertainty in the analysis will be explored using a combination of univariate and multivariate sensitivity analyses, and decision uncertainty will be addressed using cost-effectiveness acceptability curves.

## Trial status

At the time of submission of this protocol (March 2013), enrolment into the study was ongoing. Recruitment was completed in May 2013 with collection of follow-up data ongoing.

## Competing interests

The authors declare that they have no competing interests.

## Authors’ contributions

MR is involved in trial management, recruitment, and acquisition of baseline data and drafted the manuscript. AM is involved with patient recruitment and monitoring of the study. EOS oversees the health economics aspect of the study. JN oversees the statistical aspects and analysis of the study. BMcG designed the intervention, supervises the study, and contributed to editing the manuscript. All authors read and approved the final manuscript.

## Authors’ information

MR is a researcher in the Centre for Pain Research and School of Psychology at the National University of Ireland, Galway (NUI Galway). AM is Professor of General Practice at NUI Galway. EOS is Professor at School of Economics and the Centre for Social Gerontology at NUI Galway. JN is Senior Lecturer in Biostatistics at the HRB Clinical Research Facility, NUI Galway. BMcG is Senior Lecturer in Clinical Psychology and Co-Director, Centre for Pain Research at NUI, Galway and Principal Investigator of the study.
